# When Elevated Creatinine Is Not What It Seems: Intraperitoneal Urinary Leak Mimicking Acute Kidney Injury

**DOI:** 10.7759/cureus.75017

**Published:** 2024-12-03

**Authors:** Hatem Ahmed, Mohamed K Mansour, Hamza Obeid

**Affiliations:** 1 Internal Medicine, Phoenixville Hospital - Tower Health Medical Group, Phoenixville, USA; 2 Hospital Medicine, Cleveland Clinic Abu Dhabi‎, Abu Dhabi‎, ARE

**Keywords:** : acute kidney injury, intraperitoneal urinary leak, peritoneal absorption of urine, peritoneal irritation, postoperative renal failure, prostatectomy complications, renal failure and pain, urinary anastomotic leak (ual), urinoma, urinoma management

## Abstract

Acute kidney injury (AKI) is typically classified as prerenal, renal, or postrenal in etiology, with postrenal often referring to obstructive causes. However, certain uncommon conditions, such as intraperitoneal urinary leaks, may not fit clearly into these categories. In patients with a recent history of pelvic procedure, a complication such as intraperitoneal urinary leak can mimic AKI due to urine reabsorption across the peritoneum. When these leaks present beyond the immediate postoperative period, they can be challenging to diagnose, potentially leading to delayed management and complications.

We report the case of a male patient who presented with lower abdominal pain and dysuria four weeks after undergoing radical prostatectomy. His initial evaluation, including urinalysis and imaging, suggested a urinary tract infection (UTI), and he was discharged with antibiotics. Three days later, he returned to the emergency room with persistent symptoms, new-onset diarrhea, and elevated creatinine compared to baseline. He was diagnosed with AKI, presumed to be secondary to dehydration, and received intravenous (IV) fluids. Despite undergoing treatment, his symptoms worsened, with further deterioration in renal function in the absence of a clear cause. An abdominal MRI ultimately revealed a fluid collection behind the bladder, indicative of a urinoma. His symptoms and renal function improved significantly after the Foley catheter placement.

Intraperitoneal urinary leaks should be considered in post-pelvic surgery patients with unexplained serum creatinine elevations, as delayed recognition can lead to significant morbidity. This report underscores the importance of including intraperitoneal urinary leaks in the differential diagnosis for patients presenting with AKI following recent pelvic surgeries or procedures.

## Introduction

We present a case of pseudo-renal failure secondary to the delayed onset of an atypical prostatectomy complication. Acute kidney injuries (AKIs) are commonly classified as prerenal, renal, and postrenal [1]. However, in this case, the increase in serum creatinine was not related to any of these classifications. Our main objective is to highlight the diagnostic challenges associated with this condition and review the available literature on its clinical features and treatment options. This report illustrates the importance of considering uncommon causes of abnormal renal function, particularly in post-surgical patients.

## Case presentation

**Figure 1:**
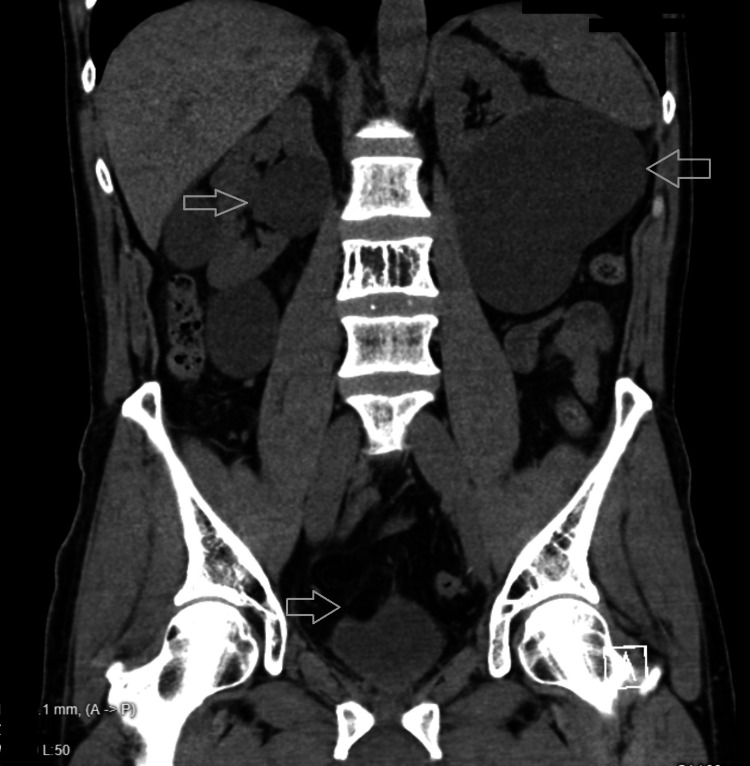
CT abdomen and pelvis without contrast (coronal view) CT: computed tomography

**Figure 2:**
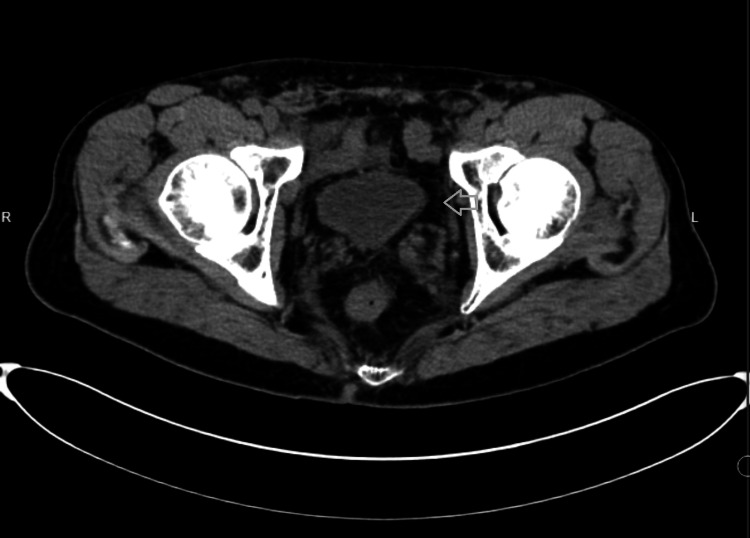
CT abdomen and pelvis without contrast (axial view) CT: computed tomography

The patient was a 65-year-old male with a history of polycystic kidney disease and prostate cancer status post laparoscopic radical prostatectomy four weeks before presentation. He presented with lower abdominal pain and burning micturition for three days. Initially, he had sought care in the emergency department, where he had been diagnosed with a urinary tract infection (UTI) based on positive urine analysis showing white blood cells (WBCs) and microscopic hematuria. A CT of the abdomen and pelvis without contrast at that time had revealed incidental findings of bilateral renal cysts and non-obstructive 2 mm stones in both kidneys, but no evidence of hydroureter, hydronephrosis, or organized collections in the pelvic region (Figures 1-2). Blood and urine cultures had been obtained, and he had been prescribed oral cephalexin and discharged from the emergency department. The urine culture had subsequently grown Streptococcus agalactiae, which was sensitive to the prescribed antibiotics. 

Despite 72 hours of compliance with antibiotics, the patient presented again to the emergency department with worsening suprapubic pain exacerbated by urination and persistent burning micturition. He also reported having had 10 loose watery stools the day before presentation but denied fever, nausea, vomiting, or other systemic symptoms. He had no history of recent nonsteroidal anti-inflammatory drugs (NSAIDs) use or intravenous (IV) contrast exposure. Physical examination was generally unremarkable, with only mild generalized abdominal tenderness on deep palpation, no rebound or guarding, and well-healed laparoscopic scars from the prostatectomy. 

Laboratory workup revealed normal complete blood count (CBC), elevated serum creatinine of 1.54 mg/dL compared to the patient’s baseline of 1.15 mg/dL, urea of 8 mmol/L, estimated glomerular filtration rate (eGFR) of 51 ml/min, and C-reactive protein (CRP) of 3 mg/L. Liver function tests were normal. Urinalysis showed clear urine with few WBCs and microscopic hematuria. Repeat Urine and blood cultures were negative. The remaining laboratory results are outlined in Table 1. The patient was admitted with a diagnosis of AKI, likely secondary to dehydration and UTI. He was treated with IV fluids (normal saline 100ml/hr) and IV antibiotics (cefoxitin 2g every eight hours) given the previously diagnosed UTI.

**Table 1:** Laboratory results of the patient ^*^Indicates abnormal lab value outside the reference range NA: not tested or not applicable

Variable	Reference range	Baseline	Day 0	Day 1	Day 2	Day 3	Day 4	Day 7	Day 14
Creatinine	0.67–1.18 mg/dL	1.15	1.54^*^	1.45^*^	2.52^*^	2.6^*^	2.13^*^	1.63^* ^	1.13
Urea	2.8–8.1 mmol/L	5.5	6.1	6.3	9.9^*^	10^*^	9^*^	7.5	5.9
Sodium	136–145 mmol/L	141	140	139	131^* ^	126^*^	130^*^	132^*^	137
Potassium	3.6–4.8 mmol/L	4	3.9	4	4.7	4.4	4.5	4.5	4.2
Hemoglobin	126–174 g/L	131	129	116^* ^	122^*^	113^*^	116^*^	128	129
White blood cells	4.5–11.0 x10^9/L	5.5	7.1	6.3	7.8	5.9	4.6	4	5.7
C-reactive protein	0.0–4.9 mg/L	0.6	3	NA	NA	131.3^*^	145.4^*^	76.9^*^	1
Procalcitonin	0–0.04 mcg/L	NA	NA	NA	NA	0.29^*^	0.25^*^	0.13^*^	NA
Lactate	0.5–2.2 mmol/L	NA	NA	NA	NA	1.3	NA	NA	NA

**Figure 3:**
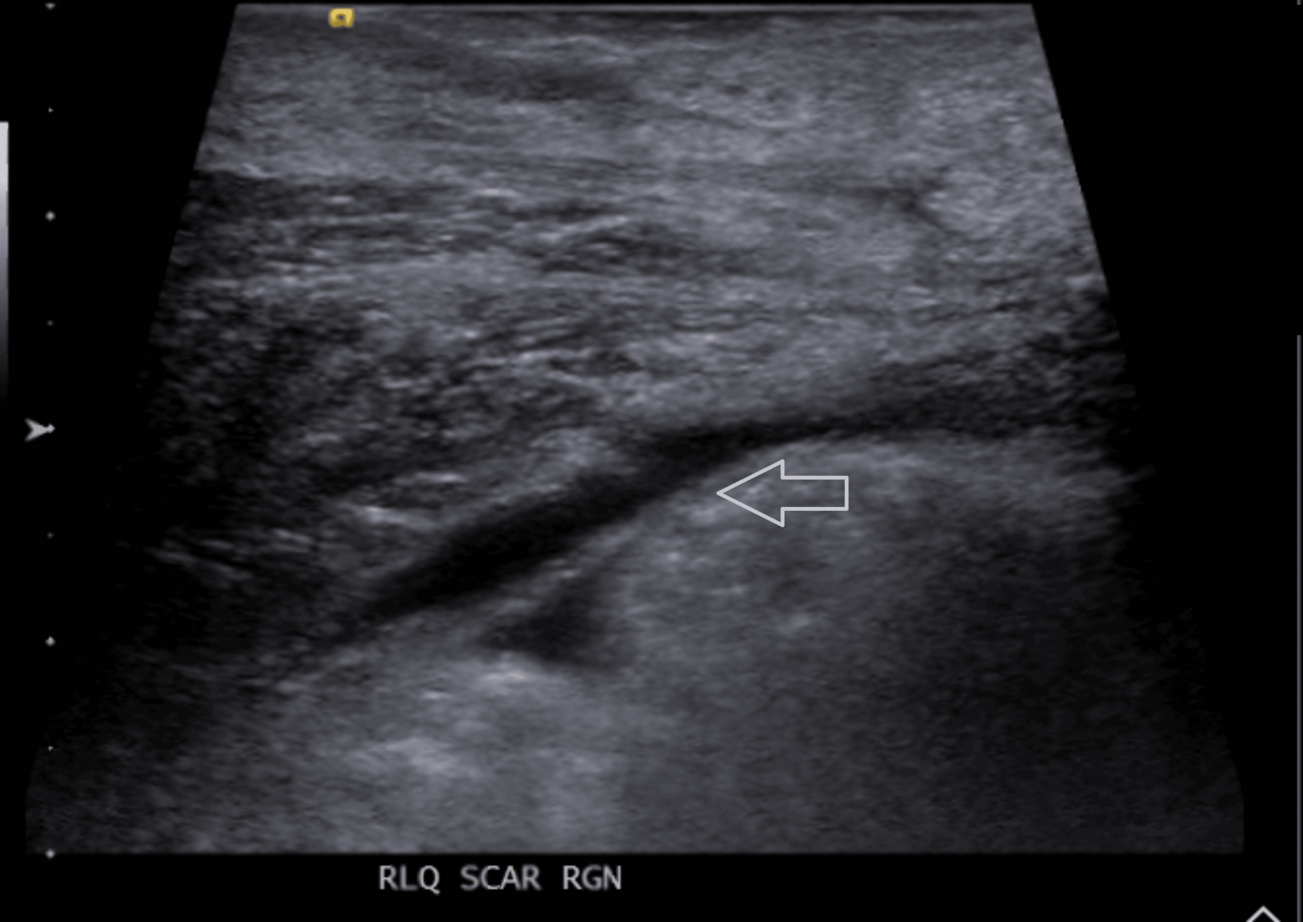
Ultrasound of the abdomen

Over the next 24 hours, the patient’s lower abdominal pain worsened significantly, particularly with urination. Physical examination showed signs of peritoneal irritation with positive rebound tenderness. Despite adequate urine output of over 3000 ml daily, his serum creatinine increased to 2.52 mg/dL. A bladder scan revealed no significant post-void residual volume and an ultrasound of the abdomen showed minimal fluid in the right lower quadrant at the surgical site, with surrounding soft tissue edema (Figure 3).

The nephrology team was consulted, who requested further urine studies: urine sodium and urine creatinine, which reported a fractional excretion of sodium of 0.17% suggestive of prerenal etiology of AKI; therefore, recommendations were made to increase the rate of IV fluids to 150 ml/hr. The urology team was consulted and they started the patient on solifenacin and phenazopyridine for cystitis-related pain. His diarrhea resolved spontaneously, and Clostridium difficile PCR was negative. Further tests requested by the nephrology team to exclude other causes of AKI included urine eosinophils, which came out to be 3% (indeterminant), and an autoimmune screen (complement 3, complement 4, antinuclear antibody, anti-glomerular basement membrane antibody, anti-myeloperoxidase antibody, and anti-proteinase 3 antibody), which came out to be negative. 

**Figure 4:**
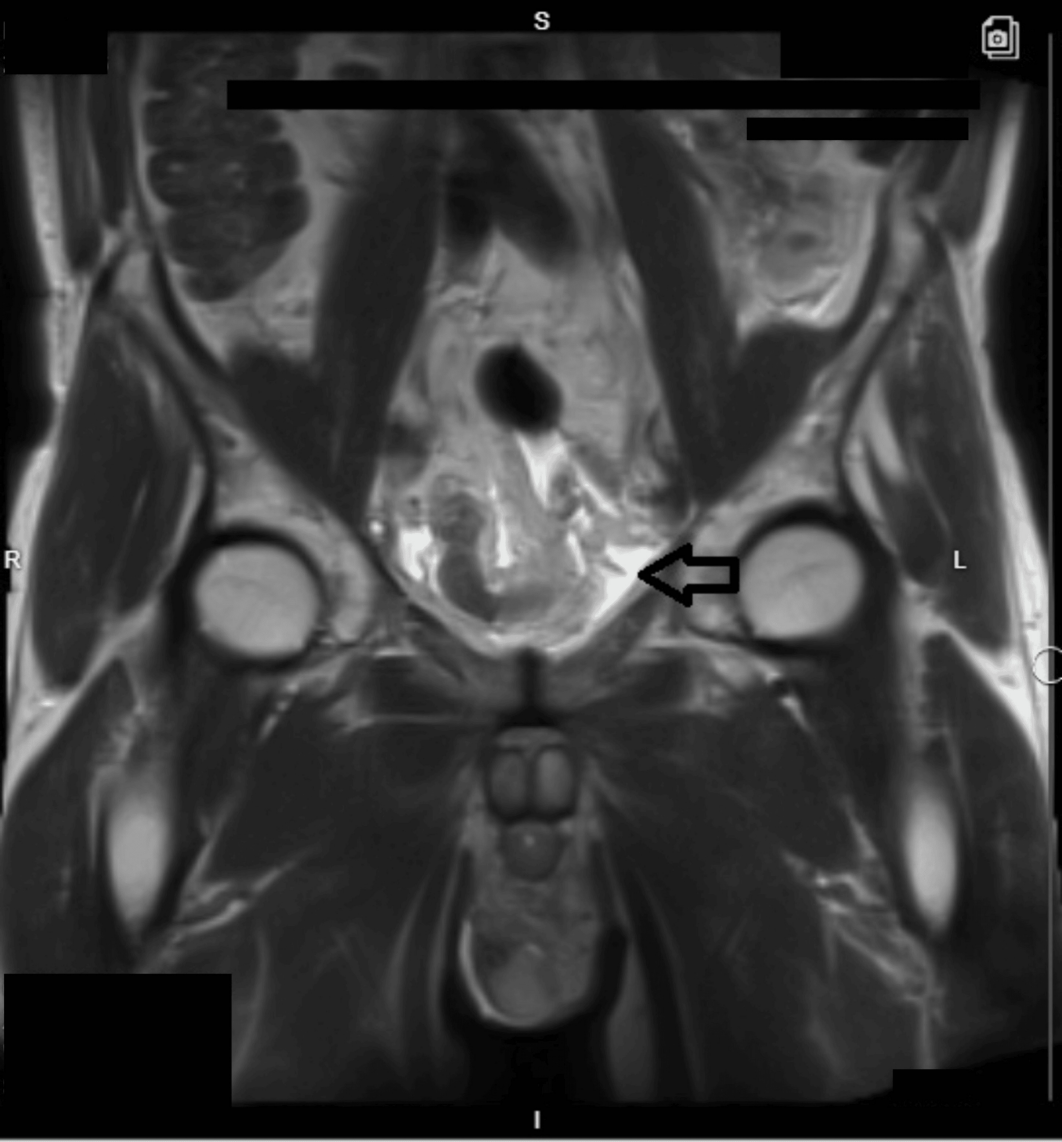
MRI of the abdomen and pelvis (T2-weighted coronal view) MRI: magnetic resonance imaging

**Figure 5:**
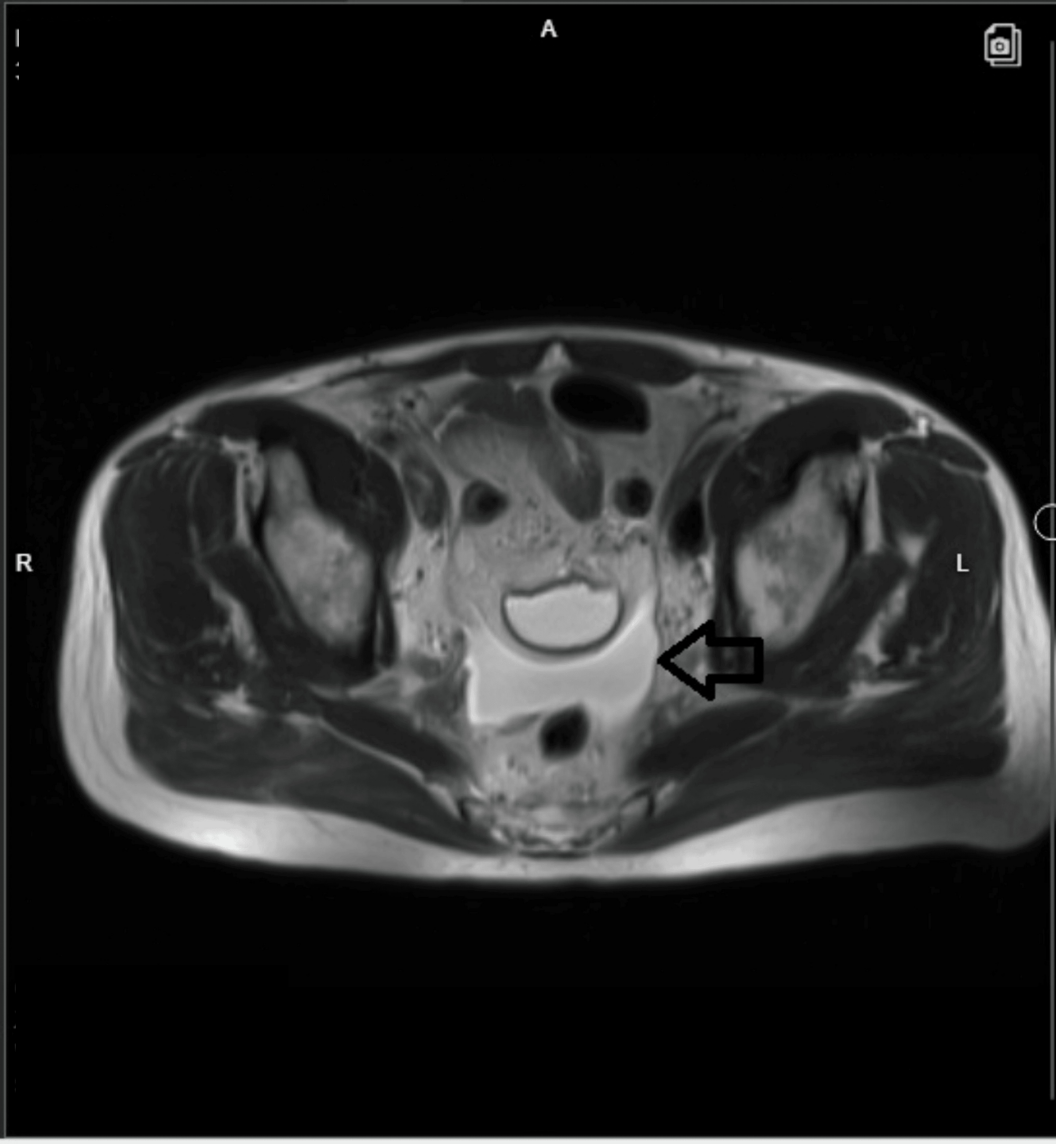
MRI of the abdomen and pelvis (T2-weighted axial view) MRI: magnetic resonance imaging

On day three of hospitalization, the patient's abdominal pain persisted; he was afebrile, and a repeat blood workup showed normal WBCs. However, CRP increased to 56.5 mg/L, procalcitonin was elevated at 0.22 mcg/L and serum creatinine increased to 2.6 mg/dL. Hence, we decided to escalate antibiotics to piperacillin/tazobactam 2.25 g every six hours (renal modified dose for GFR of 27 ml/min) and obtain an MRI of the abdomen. The MRI revealed amorphous simple-appearing fluid posterior to the urinary bladder (Figures 4-5) suggestive of a possible urinoma, likely related to the recent prostatectomy. 

The urologist inserted a Foley catheter (French size 12), which led to a gradual improvement in both abdominal pain and serum creatinine levels. The patient was discharged on day six with the Foley catheter in place, and on oral antibiotics (ciprofloxacin 250 mg BID for two weeks) to prevent bacterial translocation, and a follow-up was scheduled in two weeks for catheter removal. 

The patient presented two weeks post-discharge to the urology clinic with no further complaints of abdominal pain, and repeat serum creatinine returned to baseline. A repeat abdominal ultrasound reported no visualized free fluids in the pelvis. A retrograde urethrogram was performed, which showed no signs of contrast extravasation. Hence, the Foley catheter was removed. 

## Discussion

Urinomas are characterized by extravasation of urine into the abdominal cavity [2]. It is a reported complication post-prostatectomy that may happen due to injury at any level of the urinary collecting system. The most common cause of urine leak is vesicourethral anastomosis [3] with a reported incidence of 0.1-15.4% post-radical prostatectomy [4-5]; most cases are reported within seven days of the procedure [6]. 

Urinary leakage can lead to intraperitoneal dissemination of urine, leading to abdominal symptoms such as pain, signs of peritoneal irritation, ascites, ileus, or reduced urine output. Consequently, patients often present with poor oral intake and dehydration, along with elevated serum creatinine. This presentation can be mistaken for AKI secondary to hypovolemia; however, it may result entirely from intraperitoneal urine reabsorption or pseudo-azotemia. This occurs as solutes normally excreted in urine diffuse across the peritoneum along the concentration gradient, a process known as reverse autodialysis. Delay in presentation can exacerbate biochemical abnormalities, including elevated serum urea, creatinine, and potassium, as well as decreased serum sodium [7].

Managing AKI commonly starts with intravenous fluids after excluding obstructive causes; however, further intravenous fluids in such cases would result in more urine production and subsequently more urine leakage and reabsorption from the peritoneum, leading to further elevation in serum creatinine. In such cases, the treatment would involve reducing fluid intake and insertion of a Foley catheter to allow for healing of the vesicourethral anastomosis and prevent further leakage of urine into the peritoneum [8]. Foley catheter insertion in our patient was followed by significant improvement in his symptoms and serum creatinine.

Even though the most common cause of such a condition is traumatic or iatrogenic. There have been reports of urinary extravasation due to spontaneous bladder rupture as well [9-12]. Clinically, patients' presentations can vary from being asymptomatic to presenting with an acute abdomen. Abdominal imaging plays a crucial role in diagnosis; contrast-enhanced CT with delayed imaging, CT cystography, and retrograde urethrography are the diagnostic imaging studies of choice [13]. Significant Urinary leakages usually present with signs of peritonitis [14]. However, there are case reports of this condition without frank signs of peritonitis [15]. In patients presenting with significant ascites, measurement of peritoneal fluid urea nitrogen and creatinine can be diagnostic with values usually observed in urine samples rather than in blood [16]. 

The management of such a condition depends on the amount of leakage, mechanism of injury, electrolyte imbalance, and level of increase in serum creatinine. Urinomas that are too small to drain usually reabsorb without intervention in most cases; however, in the instance of large urinomas that can be expanding or with signs of sepsis, drainage is often required, to be performed by urology or interventional radiology. Failure to do this promptly can lead to further complications such as abscess formation and hydronephrosis [17].

## Conclusions

We discussed a case of an atypical presentation of intraperitoneal urinary leakage resembling AKI. Our findings suggest that in patients who have undergone recent pelvic procedures, the presence of urinary leak should be considered in the differential diagnosis of AKI, especially if the common causes of AKI have been ruled out. Although it is a rare condition, a delay in diagnosis can worsen the prognosis.
